# Clinical implement of Probe‐Capture Metagenomics in sepsis patients: A multicentre and prospective study

**DOI:** 10.1002/ctm2.70297

**Published:** 2025-04-03

**Authors:** Qin Sun, Ran Teng, Qiankun Shi, Yun Liu, Xing Cai, Bin Yang, Quan Cao, Chang Shu, Xu Mei, Weiqi Zeng, Bingxue Hu, Junyi Zhang, Haibo Qiu, Ling Liu

**Affiliations:** ^1^ Jiangsu Provincial Key Laboratory of Critical Care Medicine Department of Critical Care Medicine Zhongda Hospital School of Medicine Southeast University Nanjing China; ^2^ Department of Intensive Care Unit Nanjing First Hospital Nanjing Medical University Nanjing China; ^3^ Department of Critical Care Medicine The First Affiliated Hospital of Nanjing Medical University Nanjing China; ^4^ Department of Critical Care Medicine Northern Jiangsu People's Hospital Clinical Medical College Yangzhou University Yangzhou China; ^5^ Center for Infectious Diseases Vision Medicals Co., Ltd Guangzhou China

**Keywords:** antibiotics adjustment, clinical impact, diagnosis, metagenomics, sepsis

## Abstract

**Background:**

Accurate pathogen identification is critical for managing sepsis. However, traditional microbiological methods are time‐consuming and exhibit limited sensitivity, particularly with blood samples. Metagenomic sequencing of plasma or whole blood was highly affected by the proportion of host nucleic acid.

**Methods:**

We developed a Probe‐Capture Metagenomic assay and established a multicentre prospective cohort to assess its clinical utility. In this study, 184 blood samples from patients suspected of sepsis were sent for blood culture and Probe‐Capture Metagenomic sequencing before using antibiotics. The pathogen‐positive rate and auxiliary abilities in diagnosis were compared among Probe‐Capture Metagenomics, blood culture and real‐time PCR (RT‐PCR). Antibiotic therapy adjustments were based on the identification of pathogens, and changes in the Sequential Organ Failure Assessment (SOFA) score were monitored on days 0, 3 and 7 of admission.

**Results:**

A total of 184 sepsis patients were enrolled, with a mean age of 66 years (range 56–74). The Probe‐Capture Metagenomics method, confirmed by RT‐PCR, demonstrated a significantly higher pathogen detection rate than blood culture alone (51.6% vs. 17.4%, *p* < .001). When combining the results of blood culture and RT‐PCR, Probe‐Capture Metagenomics achieved a concordance rate of 91.8% (169/184), with a sensitivity of 100% and specificity of 87.1%. In terms of clinical impact, antibiotic therapy was adjusted for 64 patients (34.8%) based on the results from Probe‐Capture Metagenomics, and 41 patients (22.3%) showed a > 2‐point decrease in SOFA score following antibiotic adjustments.

**Conclusion:**

Probe‐Capture Metagenomics significantly enhances the ability of pathogen detection compared with traditional metagenomics. Compared to blood culture and RT‐PCR in sepsis patients, it leads to improved antibiotic treatment and better patient outcomes. This study, for the first time, evaluates the clinical impact of metagenomic sequencing by integrating antibiotic adjustments and SOFA score changes, indicating that approximately one‐fifth of sepsis patients benefit from this advanced diagnostic approach.

**Trial registration:**

This study has been registered in clinical trials (clinicaltrials.gov) on 30 November 2018, and the registration number is NCT03760315.

**Key points:**

- Probe‐Capture Metagenome had a significantly higher positive rate than blood culture (51.6% vs. 17.4%, *p* < .001).
- Combining blood culture and RT‐PCR results, Probe‐Capture Metagenome achieved a consistency rate of 91.8%.
- Antibiotics were adjusted in 34.8% of patients based on Probe‐Capture Metagenome results, and 22.3% of patients experienced a more than 2‐point decrease in SOFA score.

## Graphical abstract

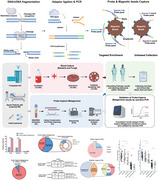

## 1 INTRODUCTION

Sepsis is a life‐threatening condition characterized by a dysregulated host response to infection, leading to organ dysfunction. Globally, sepsis continues to be a major health burden, with a morbidity of nearly 50 million and a mortality of 11 million annually.^1^ Timely administration of appropriate antibiotics is critical for improving outcomes in patients with suspected sepsis.^2^, ^3^ However, most initial treatments were based on empirical approaches, and approximately 20% of sepsis patients with bloodstream infections (BSIs) receive inappropriate or overly broad empiric antibiotics treatments, which then caused increased mortality rates.^4^, ^5^ Therefore, prompt and accurate identification of the causative pathogens is essential for implementing targeted antimicrobial therapy, reducing adverse effects and improving patient prognosis.

Culture‐based methods remain the gold standard for pathogen identification in sepsis. However, the time required for culture results and the limited sensitivity and specificity of blood cultures − ranging from 10% to 50% in patients with sepsis or septic shock − pose significant challenges, particularly for critically ill patients who may have already undergone lesion drainage or treatment.^6^, ^7^ Polymerase chain reaction (PCR)‐based assays, as culture‐independent diagnostic tools, can identify pathogens in BSI; however, PCR is limited by the need for pre‐designed primers, restricting its ability to detect only targeted pathogens.^8^ Metagenomic sequencing (mNGS) offers a less‐biased, high‐throughput method for pathogen detection and has been increasingly utilized to diagnose a wide range of infectious diseases, including BSI^9^, ^10^ and sepsis.^11^, ^12^, ^13^, ^14^, ^15^ Nevertheless, the predominance of host DNA/RNA‐often comprising over 99% of the total nucleic acid − significantly limits the sensitivity of mNGS.^16^, ^17^ One promising solution to this challenge is the use of capture probes for hybridization,^16^ which can capture specific target sequences by designed probes.^18^ The combination of hybridization‐based capture and mNGS (referred to as Probe‐Capture Metagenomic sequencing or Probe‐Capture Metagenomics) has emerged as a powerful tool for pathogen identification, though few studies have explored its application in the aetiological diagnosis of infections.^19^, ^20^

Effective pathogen identification is crucial for guiding antibiotic treatment in sepsis and the usefulness of hybridization‐based capture and mNGS brought a bridge from wide‐spectrum antibiotics to targeted antibiotic uses. While most studies have focused on the role of pathogen detection in diagnosis and antibiotic selection, fewer have examined its potential impact on clinical outcomes and prognosis.^10^, ^12^, ^15^, ^16^, ^18^ The Sequential Organ Failure Assessment (SOFA) score^21^ is widely used to assess the severity of sepsis^22^ and track organ dysfunction in critically ill patients.^23^ Although a direct correlation between SOFA score and mortality is not always evident, changes in the SOFA score (Delta SOFA) have been shown to predict outcomes and reflect treatment efficacy.^22^, ^24^, ^25^, ^26^ Given the clinical importance of pathogen identification, we hypothesize that the Delta SOFA score could serve as an indicator of the clinical impact of the Probe‐Capture Metagenomics assay in sepsis management.

To evaluate the clinical performance of Probe‐Capture Metagenomics for sepsis diagnosis and its guiding impact on therapeutic adjustment and the prognosis, we conducted a prospective, multicentre study enrolling patients with suspected sepsis, septic shock or BSI. This study systematically assessed the clinical value of Probe‐Capture Metagenomics in patients with suspected sepsis, septic shock or BSI, with a focus on its diagnostic accuracy, therapeutic impact and potential influence on patient outcomes.

## 2 MATERIALS AND METHODS

### 2.1 Study design

#### 2.1.1 Trial population

This multicentre prospective study was conducted across four medical centres in China: Southeast University Zhongda Hospital, Jiangsu Province Hospital, Nanjing First Hospital and Northern Jiangsu People's Hospital. This study was approved by the ethics committee of the central institutional review board in Southeast University Zhongda Hospital (2021ZDSYLL304‐P02), and ethics committees of other research centres were Jiangsu Province Hospital: 2022‐SR‐028, Nanjing First Hospital: KY20220314‐06 and Northern Jiangsu People's Hospital: 2022ky123. This study has been registered in clinical trials (NCT03760315). Written informed consents were provided to all selected patients. The sample collection of this study began from April 2022 and ended in November 2022.

Sepsis patients were diagnosed according to The Third International Consensus Definitions for Sepsis and Septic Shock (Sepsis‐3) (Sepsis should be defined as life‐threatening organ dysfunction caused by a dysregulated host response to infection and septic shock should be defined as a subset of sepsis in which particularly profound circulatory, cellular and metabolic abnormalities are associated with a greater risk of mortality than with sepsis alone.)^27^ and suspected BSI patients were enrolled based on the eligibility criteria. The inclusion and exclusion criteria were as follows: Inclusion criteria: (1) ICU patients had to be 18 years old or older; (2) Complied with the Sepsis 3.0 diagnostic criteria with suspected BSI and the diagnosis was made within 24 h of the disease occurrence; (3) ICU treatments were longer than 24 h; (4) Informed consents were signed by patients or their legal representatives. Exclusion criteria: (1) Patients with organ dysfunction and showed poor prognosis within 72 h after disease occurrence; (2) Patients receiving palliative care; (3) Patients without obtained informed consent. In our study, the definition of ‘Immunodeficiency’ is a condition in which the immune system's function is insufficient, leading to a reduced ability to defend the body against infections and diseases. The specific criteria are as follows: (1) Patients who are undergoing systemic immunosuppressive therapy (such as the use of corticosteroids, high‐dose immunosuppressants, chemotherapy agents or biologic agents); (2) Patients diagnosed with primary immunodeficiency or autoimmune diseases (such as systemic lupus erythematosus, rheumatoid arthritis, etc.) who are receiving immunosuppressive treatment; (3) Patients who have recently undergone organ transplantation and are receiving immunosuppressive medication; (4) Patients with HIV infection and a CD4^+^ T cell count less than 200/µL; (5) Patients with other factors, such as long‐term antibiotic therapy or other immunosuppressive states, that may affect the results of the study.

From 16 April 2022, to 20 November 2022, a total of 2927 patients were screened for eligibility upon ICU admission. Among these, 561 patients were diagnosed with sepsis and were subsequently assessed for potential inclusion in the study. After applying the study's enrolment criteria, 216 patients were ultimately enrolled in the study.

#### 2.1.2 Sample processing and data collection

Upon enrolment, blood samples were collected from each patient for blood culture, real‐time PCR (RT‐PCR) and Probe‐Capture Metagenomics assay. Additional routine microbiological tests were performed as necessary, depending on clinical indications. Demographic data, medical histories, laboratory results and microbiological test outcomes were systematically recorded. The severity of illness was assessed using the SOFA score and the Acute Physiology and Chronic Health Evaluation (APACHE) II score. Treatment regimens administered during the ICU stay were also documented, along with patient outcomes.^28^, ^29^

### 2.2 Probe‐Capture Metagenomics workflow

#### 2.2.1 Probe design for pathogen detection

The genomes of bacteria, fungi, viruses and parasites exhibit a mosaic composition, with distinct genes and genomic segments tracing unique evolutionary paths. This suggests that hierarchical trees based on overall sequence similarity may not align with those derived from individual genes.^30^ Thus, for better probe design, we used HUBDesign, a bioinformatic pipeline that crafts probes from representative sequences derived at various taxonomic levels − such as family, genus and species. This approach enables the targeting and enrichment of specific clades for the identification of both known and previously unidentified organisms. We used the default parameters of HUBDesign provided. This pipeline, which utilizes sequence homology and the versatility of DNA hybridization, has been exemplified through the creation and validation of probe sets for the comprehensive detection of all sequenced coronaviruses and for identifying bacterial pathogens implicated in sepsis.^20^ Pathogens used for probe design are listed in Table S4. The Probe‐Capture assay used in this study has gained an invention patent granted by China National Intellectual Property Administration (patent name: Group, method, kit and application of Probe Capture for detecting pathogenic microorganisms, patent No. ZL2020116367800).

#### 2.2.2 Whole blood DNA/RNA extraction, library construction, probe hybridization and sequencing

Briefly, DNA was extracted from 1 mL of whole blood samples with the QIAamp DNA Blood Mini Kit (Qiagen) as per the manufacturer's description. Using a Qubit dsDNA HS Assay Kit (Invitrogen), the isolated DNA was quantified.^31^, ^32^ A total of 400 µL whole blood samples were extracted RNA with QIAamp RNA Blood Mini Kit (Qiagen). Then, 10 µL of purified RNA was used for the cDNA library. To produce a DNA/cDNA library, the manufacturer's instructions for the KAPA low throughput library construction kit (KAPA Biosystems) were followed.^31^, ^32^ For hybrid capture‐based microbial probe enrichment, performed in one hybridization cycle, 750 ng aliquot of each sample was utilized (SeqCap EZ Library, Roche). Purification and size selection were carried out following the double‐sided bead purification procedure. A Qubit dsDNA HS assay kit was used to measure the library concentration. Library quality was assessed with an Agilent 2100 Bioanalyzer (Agilent Technologies) using a high‐sensitivity DNA kit. The library was prepared by pooling a 1.5 pM concentration of each purified sample equally for sequencing on an Illumina NextSeq 550 sequencer using a 75‐cycle single‐end sequencing strategy.

#### 2.2.3 Bioinformatic analysis

Adaptor contamination, low‐quality reads, duplicate reads and reads shorter than 40 bp were removed by Trimmomatic (v0.39).^33^ Low‐complexity reads were removed by fastp (v0.23.4) default settings.^34^ The human sequence data were identified and eliminated by mapping them to the hg38 reference genome utilizing Burrows‐Wheeler Aligner software (BWA‐0.7.17).^35^ Taxonomic classification of sequencing reads was performed using Kraken2 (v2.1.3) with default parameters including –confidence 0.2 and –threads 40. Kraken2 reference database was constructed using genomic sequences from RefSeq archaea, bacteria, viruses, plasmids, human (GRCh38), UniVec_Core, protozoa, fungi and plants, all downloaded from the NCBI RefSeq repository on 28 December 2024. To enhance microbial representation, genomes of *Pneumocystis jirovecii* (GenBank accession no. GCA_001477535.1) were manually incorporated into the database (Table S5). To mitigate spurious alignments arising from PCR duplicates or low‐complexity regions, microbial reads were subsequently aligned against the same reference database using the Burrows–Wheeler Aligner (BWA, v0.7.17) with default parameters. Only alignments exhibiting a nucleotide identity of ≥96% to the reference genome were considered valid. Furthermore, reads aligning to multiple loci within the same genus were excluded from downstream analyses to prevent ambiguous taxonomic assignments.

To account for variations in sequencing depth across samples, we normalized the sequencing reads using reads per million (RPM). Samples spiked with microorganisms were classified as positive samples and Negative Control (NC) was defined as the negative sample. The optimal positive cutoff value for each species was determined by the parameter that yielded the highest area under the curve. For microorganisms without culture isolates, the RPM mean value and standard deviation of this microorganism were calculated, and the RPM (mean + 3SD) was set as a positive cutoff value.^9^

The clinical reportable range for pathogens was established according to the following three references described in a previous study^9^: I. Johns Hopkins ABX Guide (https://www.hopkinsguides.com/hopkins/index/Johns_Hopkins_ABX_Guide/Pathogens), II. Manual of Clinical Microbiology and III. clinical case reports or research articles published in peer‐reviewed journals. All clinical reportable pathogens are listed in Table S4.

#### 2.2.4 Quality control

To monitor potential sources of contamination, plasma from healthy donors was used as the NC, and sterile deionized water was used as the Non‐Template Control. Both controls were processed in parallel with other samples in each batch.^36^, ^37^ In addition, sterile cotton swabs moistened with sterile deionized water were used to wipe the surfaces of the centrifuge and biosafety cabinet, generating a background microorganism profile for our laboratory.

#### 2.2.5 Criteria for results

Excluding viruses and non‐pathogenic normal human flora, the results of the blood Probe‐Capture Metagenomics assay were evaluated according to Figure S1. As illustrated in the figure, any discrepancies between the Probe‐Capture Metagenomics results and blood culture were subsequently re‐verified using RT‐PCR. As reference to culture or culture plus RT‐PCR, the sensitivity of Probe‐Capture Metagenomics was defined as [(sample numbers of double positive)/(sample numbers of referred assay positive)]^*^100%, while specificity was defined as [(sample numbers of double negative)/(sample numbers of referred assay negative)]^*^100%.

### 2.3 Probe‐Capture Metagenomics workflow construction and analytical performance

**FIGURE 1:**
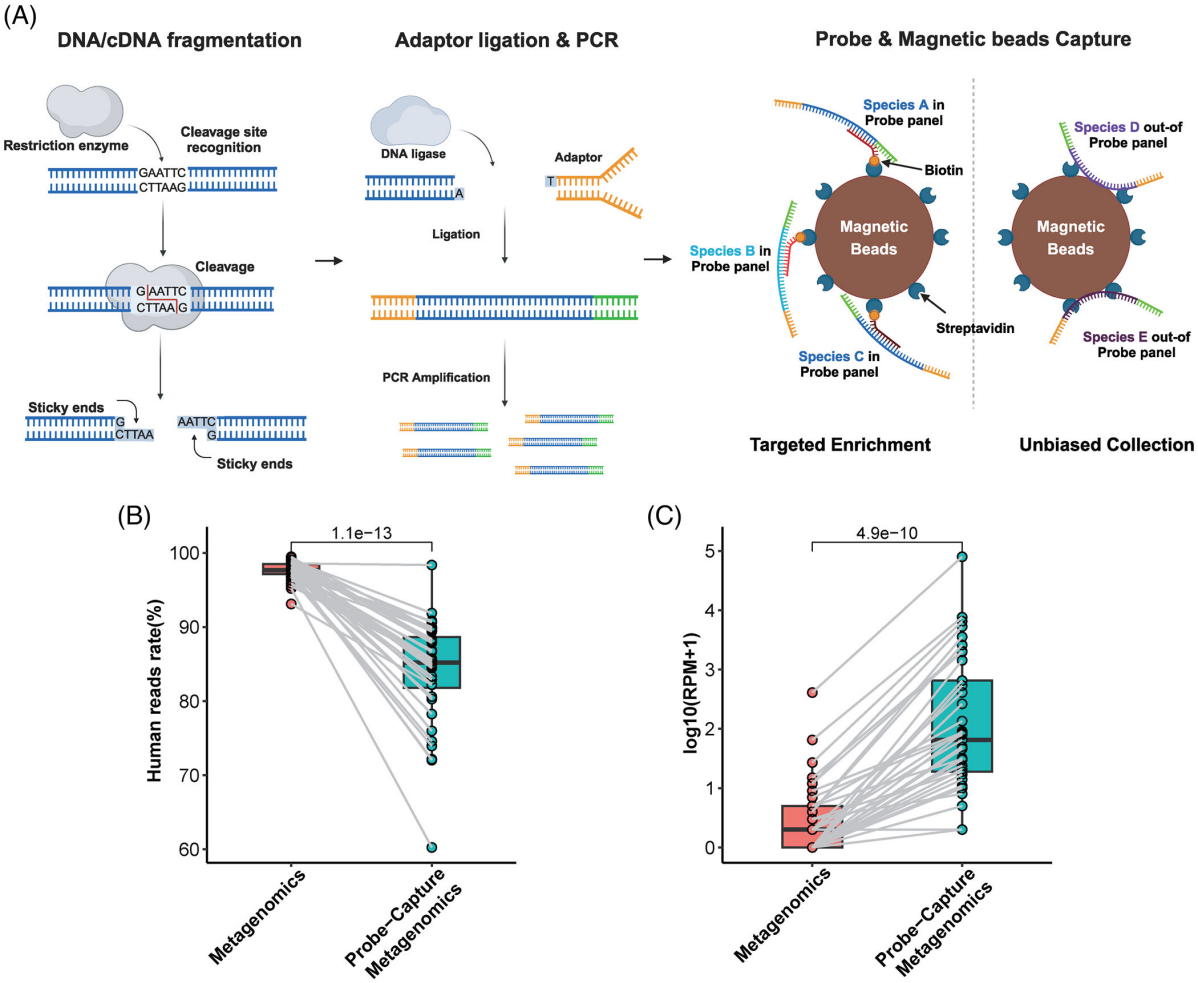
Molecular mechanism and advantages of Probe‐Capture Metagenomics in this study. (A) Molecular mechanism of Probe‐Capture Metagenomic. (B, C) Performance comparison of host depletion and pathogens enrichment between traditional metagenomics and Probe‐Capture Metagenomics. Among 184 included samples, 42 had paired traditional metagenomics results. Of these 42 samples, a total of 37 pathogens were reported by Probe‐Capture Metagenomics and also confirmed by culture or RT‐PCR. (B) Comparison of human reads rate in 40 samples sequenced by both metagenomics and Probe‐Capture Metagenomics (two samples were excluded owing to abnormal human reads rate value). (C) Comparison of reads per million (RPM) that sequenced by metagenomics and Probe‐Capture Metagenomics for 37 pathogens. Lines in box represent first quartile, median and third quartile.

The molecular mechanism of Probe‐Capture Metagenomics is illustrated in Figure 1A. According to previous studies, the cfDNA/RNA in plasma and whole blood samples is typically short,^38^ often below the preferred length of 120 bp required by Tn5 transposase for optimal library construction.^39^ Therefore, we employed endonuclease‐based library construction. Additionally, we performed targeted enrichment for key pathogens using probe hybridization and utilized the magnetic properties of beads to conduct an unbiased collection of pathogens outside the probe panel. This strategy ensures that the method we developed retains the unbiased characteristics of Metagenomics. The limit of detection (LoD), linearity and stability of the Probe‐Capture Metagenomics method were assessed for 12 representative pathogens in human Embryonic Kidney Cells 293 (HEK293T) and plasma matrices. RT‐PCR for pathogen validation was performed with the cycle threshold (Ct) value used to determine the pathogen concentration. Detailed experimental procedures and results can be found in the Supplementary Appendix (Supplementary Methods).

### 2.4 Definitions for clinical impact

We developed an algorithm integrated into the applet to automatically assess the clinical impact of Probe‐Capture Metagenomics results based on patients' microbiological test outcomes, identified pathogens, antimicrobial therapies and SOFA scores, which were recorded by the attending physician.

*A positive impact* was defined as follows: the patient's overall condition improved, at the same time the SOFA score decreased more than 2 from day 3 to day 7 after adjusting the antibiotics usage according to Probe‐Capture Metagenomics report; or the patient's overall condition got worse, the SOFA score increased more than 2 from day 3 to day 7 without adjusting antibiotics treatments according to Probe‐Capture Metagenomics results.

*A negative impact* was identified as: the patient's overall condition got worse and the SOFA score increased more than 2 from day 3 to day 7 after adjusting the antibiotics usage according to Probe‐Capture Metagenomics assay reports.

### 2.5 Statistical analysis

Continuous variables were presented as medians (first quartile and third quartile), and categorical variables were recorded as counts and percentages unless otherwise specified. The Chi‐square test was used to compare differences in categorical variables, and the Mann−Whitney U test was used for continuous variables. Data analysis was performed using GraphPad Prism 6.0 (GraphPad software). Statistical significance was considered to be present when *p *< .05.

## 3 RESULTS

### 3.1 Analytical performance characterization of Probe‐Capture Metagenomics assay

To validate the analytical performance, we conducted performance verification experiments for the Probe‐Capture Metagenomics (Supplementary Methods).^36^, ^40^ The LoD and linearity of the Probe‐Capture Metagenomics assay were evaluated by spiking 12 representative pathogens into HEK293T and plasma matrices (Table 1). The LoD for bacterial detection ranged from .06 copies/mL (*Acinetobacter baumannii*) to 238.88 copies/mL (*Staphylococcus epidermidis*) in HEK293T, and 6.73 to 3191.47 copies/mL, respectively, in plasma. For fungi, the LoD for *Cryptococcus neoformans* and *Candida albicans* was .58 and .22 copies/mL in HEK293T, and .42 and 34.55 copies/mL, respectively, in plasma. The LoD for epstein‐barr virus (EBV) was 66.54 and 29.50 copies/mL, and for cytomegalovirus (CMV), it was 177.95 and 491.82 copies/mL in HEK293T and plasma, respectively. A strong linear correlation (*R* ^2^ > .8, Figures S2 and S3) was observed between microorganism concentration and RPM value for most pathogens, with both matrices showing consistent results. Storage stability tests on microbial community samples stored at 4°C for up to 7 days showed that all microbes were correctly identified, though RPM values gradually decreased over time, particularly after 7 days (Figure S4).

**TABLE 1:** LoD for representative organisms of Probe‐Capture Metagenomics assay.

Representative organism	Type	LoD (HEK293T, copies/mL)	LoD (Plasma, copies/mL)
*Acinetobacter baumannii*	Bacterium, gram‐negative	.060415141	6.730614549
*Pseudomonas aeruginosa*	Bacterium, gram‐negative	1.890122578	115.2006898
*Klebsiella pneumoniae*	Bacterium, gram‐negative	5.67713828	23.67653447
*Escherichia coli*	Bacterium, gram‐negative	.770690159	52.1832344
*Enterococcus faecalis*	Bacterium, gram‐positive	10.83697103	260.1991731
*Salmonella enterica*	Bacterium, gram‐negative	.180757127	13.57062131
*Staphylococcus aureus*	Bacterium, gram‐positive	1.38621199	128.760787
*Staphylococcus epidermidis*	Bacterium, gram‐positive	238.8841439	3191.468375
*Cryptococcus neoformans*	Fungus	.583655819	.417005362
*Candida albicans*	Fungus	.21864369	34.55184341
EBV	DNA virus	66.54218742	29.5035307
CMV	DNA virus	177.9482455	491.8150267

Abbreviations: CMV, cytomegalovirus; EBV, epstein‐barr virus.

Comparison between traditional metagenomics sequencing and Probe‐Capture Metagenomics showed that the latter significantly reduced the ratio of human reads (85.21% [81.00%, 89.14%] vs. 97.67% [97.06%, 98.56%], *p *< .0001, Figure 1B), while enriching pathogen reads, with a significantly higher reads number (RPM) of pathogens detected in Probe‐Capture Metagenomics (*p* < .0001, Figure 1C).

### 3.2 Basic information of enrolled patients

**FIGURE 2:**
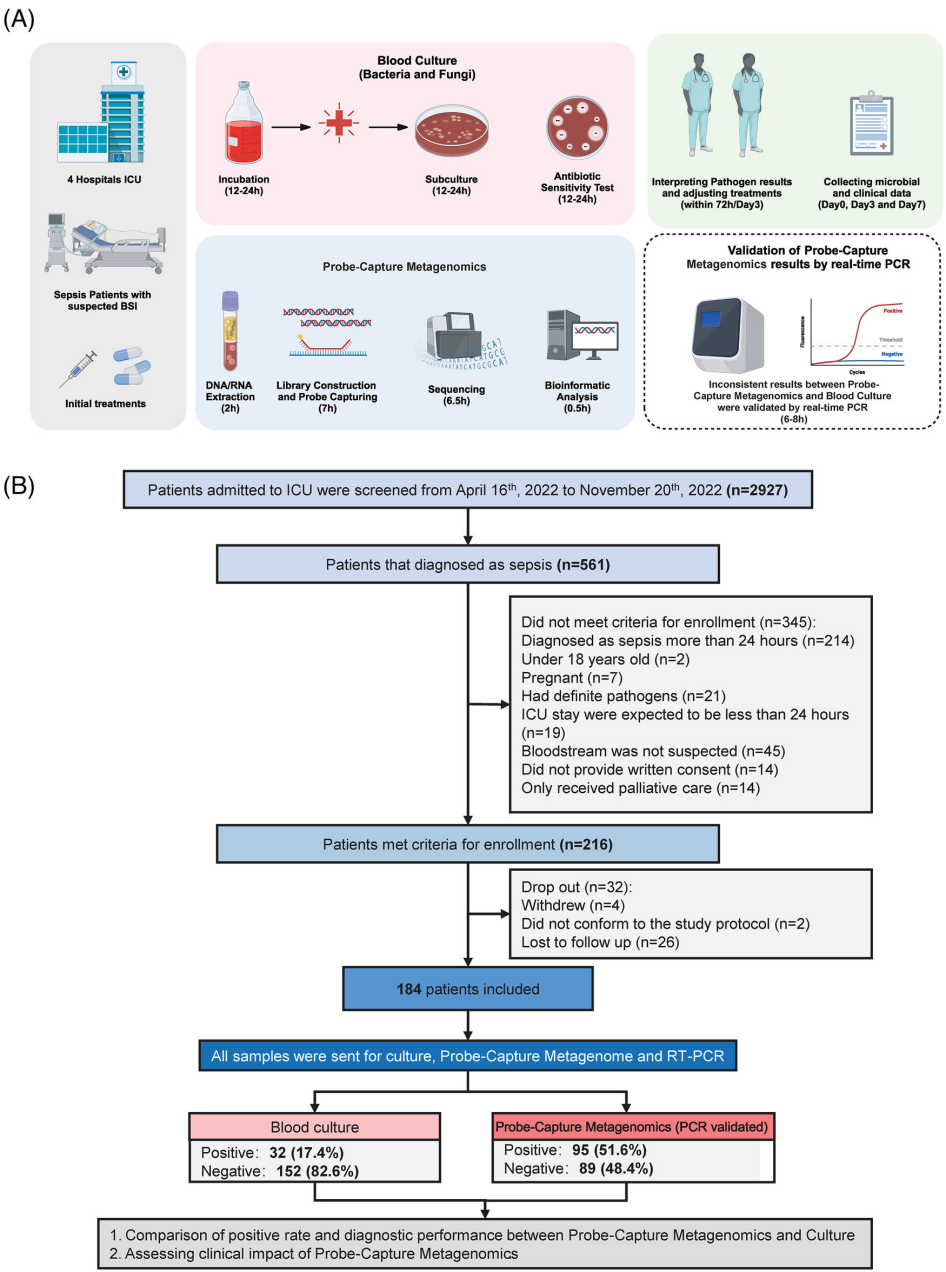
(A) Schematic of the study profile. (B) Flowchart of this study.

In addition to laboratory‐based performance verification experiments to assess the detection capabilities of Probe‐Capture Metagenomics, we established a multicentre prospective observational cohort to evaluate its application in sepsis patients with suspected BSIs. The goal of this study was to explore the potential improvement in pathogen detection rates in sepsis patients with Probe‐Capture Metagenomics, as well as to determine whether the detection of multiple pathogens contributes to changes in clinical interventions and improvements in patient prognosis (Figure 2A).

Among enrolled 216 sepsis patients, 32 dropped out in the middle, and there were 184 sepsis patients were finally included (Figure 2B). The demographic and clinical characteristics of these 184 enrolled sepsis patients are summarized in Table 2 and listed in Table S7. For all the 184 patients, the median age was 66 [56, 74] years old, 67.4% (124/184) were male and 32.6% (60/184) were female. The most common medical conditions included hypertension (45.1%), diabetes mellitus (22.3%), coronary heart disease (14.7%) and cerebral infarction (12.0%). The most common origins of infection were pulmonary, bloodstream and abdominal infections. The median SOFA and APACHE II scores at the onset of sepsis were 9 and 19, respectively. A total of 32 (17.4%) patients showed immunodeficiency, 127 (69%) patients underwent mechanical ventilation and the 28‐day mortality rate was 23.4% (43/184).

**TABLE 2:** Demographic and clinical characteristics of patients.

Characteristics	Total
**Number of patients**	184
**Gender, male, *n* (%)**	124 (67.39)
**Age, years**	66 (56, 74)
**Medical history, *n* (%)**	
Hypertension	83 (45.11)
Cerebral infarction	22 (11.96)
Diabetes mellitus	41 (22.28)
Coronary heart disease	27 (14.67)
COPD	3 (1.63)
Chronic cardiac insufficiency	7 (3.8)
CKD	10 (5.43)
Malignant tumour	16 (8.7)
Autoimmune disease	16 (8.7)
**Immunodeficiency, *n* (%)**	32 (17.39)
**Primary infectious sites**	
Lung	74 (40.2)
Bloodstream	34 (18.5)
Abdomen	33 (17.9)
Urinary tract	16 (8.7)
Liver and gallbladder	14 (7.6)
Skin and soft tissue	7 (3.8)
Intracalvarium	2 (1.1)
Other	4 (2.2)
**Laboratory examination**	
WBC − day0, 10^9^/L	11.93 (7.79, 17.24)
WBC − day3, 10^9^/L	10.09 (6.85, 14.43)
Neutrophil count − day0, 10^9^/L	10.11 (6.25, 15.58)
Neutrophil count − day3, 10^9^/L	8.62 (5.51, 12.92)
CRP − day0, mg/L	127.82 (53.57, 191.09)
CRP − day3, mg/L	89.48 (35.47, 132.16)
PCT − day0, µg/L	4.71 (.78, 23.87)
PCT − day3, µg/L	2.25 (.49, 7.16)
**Severity and outcome**	
Mechanical ventilation, *n* (%)	127 (69.02)
SOFA − day0	9 (7, 12)
SOFA − day3	7 (5, 11)
SOFA − day7	5 (3, 9)
APACHEII − day0	19 (15, 24)
APACHEII − day3	15 (12, 20)
APACHEII − day7	14 (10.5, 20)
28‐day mortality, *n* (%)	43 (23.37)
Hospitalization mortality, *n* (%)	10 (5.43)

*Note*: Continuous variables were presented as medians (first quartile, third quartile) and categorical variables as counts and percentages, unless otherwise specified.

Abbreviations: CKD, chronic renal disease; COPD, chronic obstructive pulmonary disease.

### 3.3 Positive rate comparison between Probe‐Capture Metagenomics and blood culture assays

**FIGURE 3:**
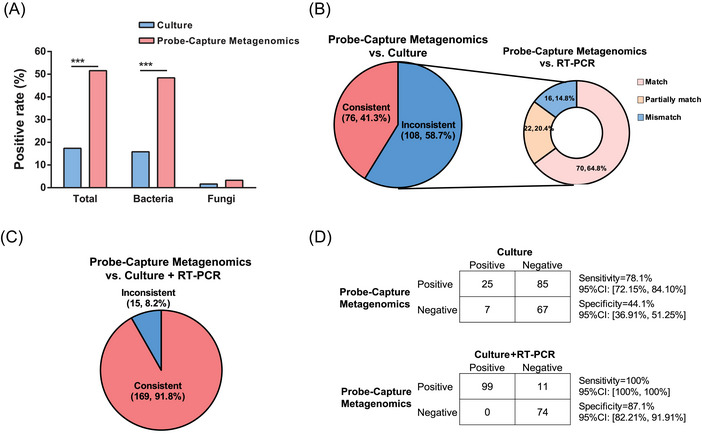
Comparison of positive rate between blood Probe‐Capture Metagenomics and blood culture. (A) The comparison of total, bacteria and fungi positive rates between blood culture and Probe‐Capture Metagenomics. ^***^ *p *< .001. (B) Pie chart demonstrating the result consistency of Probe‐Capture Metagenomics and culture. The inconsistent samples were further validated by PCR and the results were categorized as match, mismatch and partially match. Consistent: double negative (*n* = 67), double positive and detected pathogens were identical (*n* = 9); inconsistent: only Probe‐Capture Metagenomics positive (*n* = 85), only culture positive (*n* = 7), double positive but at least one pathogen does not overlap (*n* = 16); Match: double negative (*n* = 7), double positive and detected pathogens were identical (*n* = 63); Partially match: double positive and at least one overlap of pathogens was observed (*n* = 22); Mismatch: only Probe‐Capture Metagenomics positive (*n* = 15), double positive but no overlap of detected pathogens (*n* = 1). (C) After combining the results of culture and PCR, pie chart demonstrating the result consistency of Probe‐Capture Metagenomics and culture plus PCR. Consistent: double negative (*n* = 74), double positive and at least one overlap of pathogens was observed (*n* = 95); inconsistent: only Probe‐Capture Metagenomics positive (*n* = 11), double positive but no overlap of detected pathogens (*n* = 4). (D) 2 × 2 contingency tables comparing the performance of Probe‐Capture Metagenomics versus blood culture and PCR.

Blood samples from all 184 sepsis patients in this study were subjected to blood culture, RT‐PCR and Probe‐Capture Metagenomics. The results of Probe‐Capture Metagenomics were analysed in combination with RT‐PCR results according to Figure S1. When there was inconsistency between the results from Probe‐Capture Metagenomics and blood culture, RT‐PCR was employed to validate the results of both Probe‐Capture and blood culture. Among all the 184 sepsis cases, the overall positive rate for blood culture was 17.4% (32/184), whereas the positive rate for Probe‐Capture Metagenomics was 51.6% (95/184), which was considerably higher than blood culture (Figure 3A, *p *< .001). In addition, the positive rate of Probe‐Capture Metagenomics for bacteria was 48.4% (89/184), which was also significantly higher than that from blood culture, whereas the positive rate from blood culture was 15.8% (29/184; Figure 3A, *p *< .001). No significant difference about fungal detection rate was observed between Probe‐Capture Metagenomics and blood culture.

Among all 184 sepsis patients, 76 sepsis patients (41.3%) had consistent results about pathogen detection by using Probe‐Capture Metagenomics assay and blood culture (Figure 3B and Figure S5A). Subsequently, RT‐PCR data were analysed in the remaining 108 sepsis patients in order to check the discrepancy data between the Probe‐Capture Metagenomics assay and blood culture. In these 108 sepsis patients, there were 70 patients (64.8%, 70/108) showed consistent matched pathogens results between Probe‐Capture Metagenomics and RT‐PCR. In these 70 sepsis patients, there were 63 cases showed double‐positive results and 7 cases showed double‐negative results (Figure 3B and Figure S5B).

When consolidating the results from both culture and RT‐PCR, Probe‐Capture Metagenomics demonstrated an overall consistency rate of 91.8% (169/184) was observed for Probe‐Capture Metagenomics (Figure 3C and Figure S5C). Consequently, by using blood culture as a reference, Probe‐Capture Metagenomics exhibited a sensitivity of 78.1% and a specificity of 44.1%. But notably compared to RT‐PCR, the sensitivity of Probe‐Capture Metagenomics increased to 100%, although the specificity of Probe‐Capture Metagenomics remained relatively lower. However, upon combining the results of culture and RT‐PCR, the sensitivity and specificity of Probe‐Capture Metagenomics improved to 100% and 87.1%, respectively (Figure 3D).

### 3.4 Distribution of detected pathogens and correlation to clinical outcome

**FIGURE 4:**
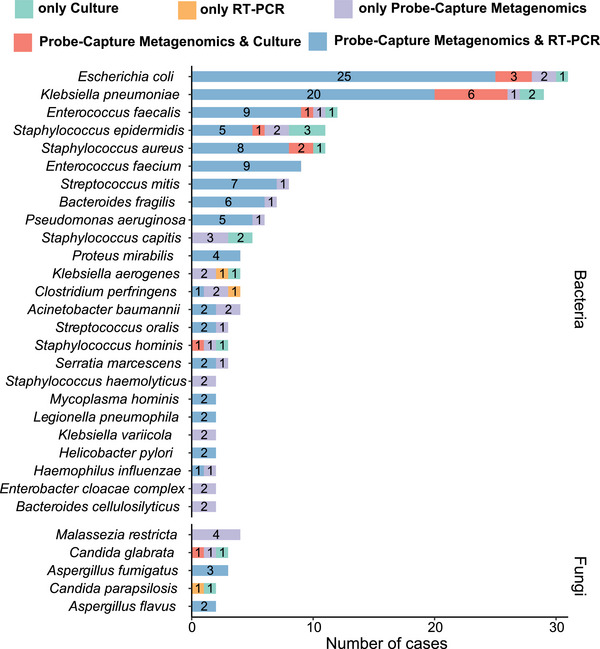
Distribution of detected pathogens comparison by Probe‐Capture Metagenomics versus culture and RT‐PCR. Bacteria and fungi identified by Probe‐Capture Metagenomics, culture, RT‐PCR or both. Pathogens are listed in order of the total detected frequency in all samples.

**FIGURE 5:**
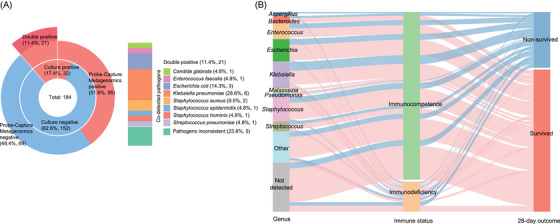
Distribution of detected pathogens and correlation to clinical outcome. (A) Comparison of the results and pathogens consistency between two groups by Probe‐Capture Metagenomics and culture, deep red sector represents double positive results. (B) Sankey diagram illustrates the relationship between positive pathogens at genus level detected by culture or Probe‐Capture Metagenomics and corresponding patients’ immune status and 28‐day outcome.

The positivity rates for pathogen detection varied between different methods and microorganisms. Most bacterial pathogens were detected by both Probe‐Capture Metagenomics and RT‐PCR, while some microorganisms were identified exclusively by metagenomic sequencing (Figure 4). The blood culture and Probe‐Capture Metagenomics (RT‐PCR‐validated) assays yielded 17.4% (*n* = 32) and 51.6% (*n* = 95) positive results, respectively, as previously mentioned (Figure 3A). Among these cases, 21 samples exhibited double positivity in both culture and Probe‐Capture Metagenomics assays (Figure 5A). The co‐detected pathogens in these 21 double‐positive samples included *Klebsiella pneumoniae* (*n* = 6), *Escherichia coli* (*n* = 3), *Staphylococcus aureus* (*n* = 2), *Enterococcus faecalis* (*n* = 1), *Candida glabrata* (*n* = 1), *S. epidermidis* (*n* = 1), *Streptococcus pneumoniae* (*n* = 1) and *Staphylococcus hominis* (*n* = 1). Conversely, the remaining five cases demonstrated inconsistencies in pathogen identification (Figure 5A).

Regarding the identified pathogens and their corresponding host patients’ 28 days outcomes, 80% (4/5) of detected *Aspergillus* cases were found in patients who could not survive and died, the majority of *Bacteroides* (70%, 7/10), *Enterococcus* (66.7%, 14/21), *Escherichia* (71.0%, 22/31), *Klebsiella* (63.9%, 23/36), *Malassezia* (100%, 5/5), *Pseudomonas* (66.7%, 4/6), *Staphylococcus* (69.7%, 23/33) and *Streptococcus* (64.3%, 9/14) were detected in sepsis patients who finally survived (Figure 5B). Additionally, among the 67 cases whose pathogenic microbes were not detected, 80.6% (54/67) of those patients finally survived (Figure 5B). For patients with immunodeficiency (*N* = 32), *K. pneumoniae* (*n* = 4) was the most frequently detected pathogens (Figure S6A). Furthermore, immunodeficient cases were confronted with significant detection of fungi, especially for *Aspergillus* (3/32 = 9.4%  vs. 2/152 = 1.3% for immunocompetence, *p *< .05), and also showed a higher rate of 28‐day non‐survival than immunocompetent patients (12/32 = 37.5%  vs. 31/152 = 20.4%, *p *< .05, Figure 5B).

Among the 28‐day non‐survivors (Figure S6B), *Klebsiella* was identified in 13 cases, followed by *Staphylococcus* (*n* = 10), *Escherichia* (*n* = 9), *Enterococcus* (*n* = 7), *Streptococcus* (*n* = 5), *Aspergillus* (*n* = 4), *Bacteroides* (*n* = 3) and other microbial genera (*n* = 12). Regarding the 28‐day mortality rates associated with specific pathogens (Figure S6C), *Aspergillus fumigatus*, *C. glabrata* and *S. hominis* exhibited a mortality rate of 66.7%, followed by *Staphylococcus capitis* (60%, 3/5), *K. pneumoniae* (37.9%, 11/29) and *Streptococcus mitis* (37.5%, 3/8). *E. faecalis* (4/12), *Enterococcus faecium* (3/9) and *Pseudomonas aeruginosa* (2/6) displayed a mortality rate of 33.3%.

### 3.5 The clinical impact of Probe‐Capture Metagenomics

**FIGURE 6:**
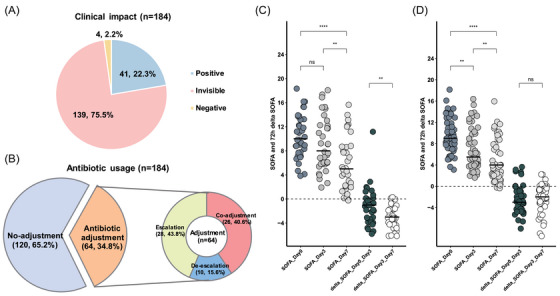
The clinical impact of Probe‐Capture Metagenomics on outcome and treatment in this study. (A) The distribution of clinical impact caused by Probe‐Capture Metagenomics on all patients. (B) The distribution of impact caused by Probe‐Capture Metagenomics on antibiotics usage, the antibiotic adjustment group was further categorized as escalation, de‐escalation and co‐adjustment. (C) Comparison of SOFA and delta SOFA among patients with positive impact. (D) Comparison of SOFA and delta SOFA among patients whose empirical therapies were confirmed by Probe‐Capture Metagenomics and kept unchanged. ^**^ *p *< .01; ^****^ *p *< .0001; ns, no significant difference.

Among the 184 sepsis patients, Probe‐Capture Metagenomics showed a positive impact on 41 patients (22.3%), no visible impacts on 139 patients (75.5%) and a negative effect on 4 patients (2.2%, Figure 6A). To better understand the clinical differences between patients who benefited from Probe‐Capture Metagenomics and those who did not, we conducted a comparative analysis of their characteristics. We found that patients with a positive impact had significantly higher SOFA scores at both day 0 and day 3 compared to patients who did not experience a positive impact (Table S1).

Antibiotics adjustments were made based on Probe‐Capture Metagenomics results. Probe‐Capture Metagenomics prompted adjustments in empirical antibiotic therapy for 34.8% of patients (64/184). Among these cases, 28 sepsis patients had antibiotic escalation, 10 patients had de‐escalation and 26 patients experienced co‐adjustment of antibiotics (Figure 6B). In contrast to patients who did not undergo antibiotics adjustments, those who had antibiotics adjustments displayed higher SOFA scores at day 0, day 3 and day 7, along with increased 28‐day mortality rates (Table S2).

For patients who experienced a positive impact, the SOFA score on day 7 was significantly lower than on day 0 and day 3 (*p* < .05, Figure 6C), and the decrease in SOFA from day 3 to day 7 was significantly greater than that from day 0 to day 3 (*p* < .05, Figure 6C), but there was no significant difference between the SOFA scores on day 0 and day 3. Conversely, for sepsis patients whose empirical antibiotics treatments were confirmed by Probe‐Capture Metagenomics and remained unchanged, no significant difference was found in the 72‐h delta SOFA between day 0 and day 3, day 3 and day 7 (Figure 6D), suggesting that the empirical treatments were effective.

## 4 DISCUSSION

This is the first prospective study to utilize Probe‐Capture Metagenomics for diagnosing suspected BSIs in sepsis patients. Due to factors such as large blood sample volumes, low pathogen concentrations, high host DNA content and fragmented cfDNA/RNA (with lengths below 70 bp), traditional microbial tests and conventional metagenomic sequencing require further optimization.^9^, ^38^ As reported by previous studies, the average nucleic acid reads derived from human account for more than 90% in traditional mNGS.^41^, ^42^ In this study, we combined probe capture with magnetic bead‐based enrichment, which not only enhanced the enrichment efficiency of key pathogens but also preserved the characteristics of metagenomics to some extent. Our Probe‐Capture Metagenomics showed a median human reads rate of 85.21%, remarkably lower than traditional metagenome. Meanwhile, the performance of pathogens enrichment was also significantly improved. As the detection limit is dependent on pathogens load, host genetic material and sequencing depth,^43^ the Probe‐Capture Metagenomics allowed for the detection of representative organisms from plasma in amounts as small as 6.73 copies/mL for bacteria, .42 copies/mL for fungi and 29.50 copies/mL for virus, significantly superior than previously reported metagenomic sequencing,^40^, ^44^ and even comparable with RT‐PCR and droplet digital PCR.^45^, ^46^ Additionally, through rigorous prospective clinical research, we statistically analysed and evaluated the clinical value of this methodology. This is a novel translational medicine study that also offers new insights for research on infection issues in ICU patients.

This prospective observation study included 184 septic patients presumed to have BSI. Through comprehensive analysis of results derived from blood culture and RT‐PCR, we observed a notable consistency rate of 91.8% between Probe‐Capture Metagenomics and the combination of culture and RT‐PCR together. Moreover, Probe‐Capture Metagenomics displayed a markedly higher sensitivity compared to culture alone. Additionally, our findings indicated that Probe‐Capture Metagenomics prompted antibiotic adjustments in 34.8% of patients, with 22.3% experiencing significant benefits defined by a noteworthy decrease in SOFA scores.

Currently, blood culture remains the gold standard for diagnosing BSIs. However, blood culture has a prolonged incubation period and relatively low positivity rate, which makes the rapid and accurate diagnosis of pathogens very challenging.^47^, ^48^ Various post‐culture technologies, such as PCR and mNGS, have emerged as new diagnostic tools which are quicker and more sensitive. Herein, our Probe‐Capture Metagenomics platform is capable of completing analysis from sampling to the final reports in 24 h, whose turnaround time was significantly shorter than that of blood culture (19.1 h [17.4−21.1 h] vs. 111.0 h [88.0−127.4 h], *p* < .001). In clinical diagnosis, specific PCR can complete the identification of previously known pathogens within 12 h.^49^ Though PCR has been shown to be more rapid and sensitive than mNGS in blood, a broader range of potential pathogens can be detected by mNGS.^37^ We validated Probe‐Capture Metagenomics results using RT‐PCR and found a high confirmation rate, highlighting its accuracy in detecting pathogens. Probe‐Capture Metagenomics outperformed blood culture with a significantly higher positive rate. Compared to composite microbiological tests (culture and PCR), its specificity improved to 87.1%. One retrospective study demonstrated a lower sensitivity of 30.2% and a higher specificity of 99.5% from plasma metagenome as compared to PCR in patients with sepsis.^37^ In our study, due to the higher sensitivity and broader detection range of Probe‐Capture Metagenomics, more false positives were observed when PCR and culture were used as the standard. This suggests that the Probe‐Capture Metagenomics method may have higher sensitivity but lower specificity. Additionally, we compared our reagent expenses with those reported in a published metagenomic study by Ramachandran et al.^50^ Our overall reagent cost is approximately $200 (Table S3). When comparing traditional metagenomics and Probe‐Capture Metagenomics, the pathogen RPM increases nearly 100‐fold (Figure 1), significantly reducing the required sequencing volume. However, despite this advantage, the overall cost of Probe‐Capture Metagenomics is not significantly lower than that of traditional metagenomics due to the added expense of probe capture. Nevertheless, with the large‐scale adoption of this technique, we are confident that reagent costs will decrease over time, ultimately making this approach more cost‐effective and providing greater benefits to patients.

Our study also revealed one of the highest pathogen identification rates among similar studies, with the most frequently detected pathogens being *Klebsiella* (*n* = 13), *Staphylococcus* (*n* = 10) and *Escherichia* (*n* = 9), which were consistent with data from China Antimicrobial Surveillance Network^51^ (CHINET, www.chinets.com). For sepsis patients, different pathogens caused different clinical outcomes. In this study, the top three causative bacteria associated with the highest mortality rates were *S. capitis*, *K. pneumoniae* and *S. mitis*. Although there was not sufficient mortality data available for bacteraemia caused by *S. capitis* and *S. mitis*, the *K. pneumoniae* bacteraemia caused mortality rate in this study aligned with previous research reports.^52^

Furthermore, our work prospectively and comprehensively considered whether Metagenomics guided antimicrobial therapy prescription along with changes in SOFA score to adjudicate the real‐world clinical impact of Metagenome. We demonstrated that Probe‐Capture Metagenomics contributed to antibiotic adjustments in 34.8% (64/184) and a positive effect in 22.3% (41/184) of septic patients. In terms of the clinical impact of mNGS in sepsis, previous studies reported remarkably high positive clinical effects of 76.3%^53^ and 71.4%.^54^ We believe they overstated the positive effects of mNGS, as their broad criteria for defining a positive impact − such as contributing to the identification of causative pathogens or confirming empirical antibiotic therapies − likely led to overly optimistic results. Previous studies have reported relatively low positive impact rates for traditional mNGS − 7.3%,^55^ 14%^56^ and 21.0%^57^ across different patient populations. Moreover, they defined clinical impact solely in terms of antibiotic treatment adjustments without patient prognosis. In contrast, our study demonstrates a higher positive impact of mNGS by evaluating actual clinical outcomes in addition to treatment modifications. We believe that confirmation of the empiric treatment or pathogen diagnosis alone does not adequately justify the necessity of mNGS. The true ‘positive impact’ should have a favourable effect on prognosis and outcome.

SOFA score of ICU patients is a good indicator for prognosis, and an increase in SOFA score during the first 48 h indicates a higher mortality rate.^28^ In randomized controlled trials, 72 h delta SOFA score was shown to be reliably and consistently associated with mortality,^58^, ^59^ which has been successively used to serve as an endpoint to evaluate the efficacy and safety of one therapy among patients with septic shock.^55^, ^60^ In this context, we prospectively employed the 72‐h delta SOFA as the fundamental criterion for Probe‐Capture Metagenomics, expectedly to yield a semi‐quantitative real‐world assessment of its clinical impact. Such an approach is valuable for clinicians to consider about the patients’ prognoses and our study strongly confirmed the favourable impact of Probe‐Capture Metagenomics on the clinical outcome of sepsis patients. However, it is worth noting that, as all patients in this study underwent both mNGS and blood culture testing, no grouping into mNGS and non‐mNGS cohorts was performed during the study design. Therefore, the observed impact of mNGS on SOFA scores and clinical outcomes was only exploratory. Further rigorous studies with appropriate grouping are needed to validate the influence of mNGS on clinical outcomes in sepsis patients.

While this study benefited from a multicentre design across four ICUs, it does have some limitations. First, we did not directly compare Probe‐Capture Metagenomics with traditional mNGS, as traditional mNGS might obscure the benefits of Probe‐Capture Metagenomics. Second, although we made a significant effort to evaluate the clinical effects of Probe‐Capture Metagenomics on sepsis patients by delta SOFA, there was still a gap between delta SOFA and real clinical outcomes. Further studies are needed to explore the effects of Probe‐Capture Metagenomics on patients’ prognoses. Third, this study included sepsis patients with relatively mild disease severity, which may introduce bias into the results and limit the generalizability of the conclusions to patients with more severe sepsis. Fourth, although, we adhered to a standardized protocol mandating that blood cultures be drawn prior to the initiation of empirical antibiotic therapy within our institution, it was still unavoidable that some patients had received antibiotics before admission. Finally, a single patient sample might detect multiple pathogenic microorganisms, and the determination of the causative pathogen primarily relied on the physicians’ decision. This could potentially lead to biases in the interpretation of test results and subsequent treatment adjustments.

## 5 CONCLUSION

In conclusion, this multicentre prospective study demonstrated that the Probe‐Capture Metagenomics assay was a highly sensitive, broad‐spectrum method for pathogen detection, exhibiting strong concordance with RT‐PCR and superior sensitivity and turnaround time compared to blood culture. Furthermore, we highlighted the positive clinical impact of Probe‐Capture Metagenomics assay on sepsis patients by using the delta SOFA score. Our findings provide a comprehensive, semi‐quantitative assessment of the assay's diagnostic performance and clinical utility in the management of sepsis.

## AUTHOR CONTRIBUTIONS

LL and HBQ had full access to all of the data in the study and took responsibility for the integrity of the data and the accuracy of the data analysis. Concept and design: QS, LL and BY; Acquisition, analysis or interpretation of data: RT, QKS, YL, XC, QC and CS; Drafting of the manuscript: QS, BY and LL; Critical revision of the manuscript for important intellectual content: LL and HBQ; Statistical analysis: XM, WZ, BH and JZ; Administrative, technical or material support: BY, XM, WZ and BH.

## CONFLICT OF INTEREST STATEMENT

The authors have no potential conflicts of interest.

## FUNDING

This work was supported, in part, by the National Key Research and Development Program of China (2022YFC2504405), the National Natural Science Foundation of China (82341032, 81930058, 82270083), the Second Level Talents of the ‘333 High Level Talents Training Project’ in the sixth phase in Jiangsu (LGY2022025), and Jiangsu Provincial Medical Key Laboratory (ZDXYS202205) and ‘Hongmian Plan’ Project of Guangzhou (No. HMJH‐2020‐0005).

## ETHICS APPROVAL AND CONSENT TO PARTICIPATE

This study was approved by the ethics committee of the central institutional review board in Southeast University Zhongda Hospital (2021ZDSYLL304‐P02) and ethics committees of other research centres (Jiangsu Province Hospital: 2022‐SR‐028, Nanjing First Hospital: KY20220314‐06 and Northern Jiangsu People's Hospital: 2022ky123). Written informed consent was provided by all enrolees or surrogates.

## Supporting information



Supporting Information

Supporting Information

## Data Availability

The datasets generated and/or analysed during the current study are not publicly available considering the privacy or ethical restrictions but are available from the corresponding author on a reasonable request. Microbial sequencing data are available at NCBI Accession no. PRJNA1078115.
