# Supplementary material for: Clinical implement of Probe‐Capture Metagenomics in sepsis patients: A multicentre and prospective study
Source: Clin Transl Med. 2025 Apr 3;15(4):e70297. doi: 10.1002/ctm2.70297 (PMC11968419; doi:10.1002/ctm2.70297)
Supplement: Supplementary file 1 — Supporting Information [file CTM2-15-e70297-s001.docx]

**Supplementary Appendix**

**Clinical implement of Probe-Capture Metagenomics in sepsis patients: a multicenter and prospective study**

**Supplementary Methods**

**Limit of detection (LoD)**

An LoD value was estimated for each of the 12 representative pathogens in HEK293T and plasma matrix, respectively. For the cellular DNA method, all representative pathogens were spiked into HEK293T cells matrix, and for the cfDNA method, a matrix of plasma pooled from healthy donors was used to dilute sheared DNA from all representative pathogens. A series of log dilutions from 1:1 to 1:2187 of pathogens was generated and each concentration was tested using Probe-Capture Metagenomics with five replicates. The LoD was calculated using probit regression analysis as the concentration at which organism was successfully detected in 95% of replicates, with five replicates performed at each tested concentration.

**Linearity**

The linearity was also evaluated by performing a linear regression analysis on the same five sets of serially diluted samples used in LoD assay. The log10-transformed RPM values were plotted against the log10-transformed total input organism concentration (copies/mL). The best-fit regression line, along with the linear equation and R^2^ value, were added to the plotted values using the R package.

**Stability**

To evaluate the stability of the assay, five replicates of samples were thawed and placed in a refrigerator at 4°C for 0, 4 and 7 days. After generating metagenomics libraries from the samples and sequenced, the acquired RPM for each microbe was compared among the various storage duration.

**Real-time PCR for pathogen validation**

For detection of pathogens in blood specimens, a real-time polymerase chain reaction (RT-PCR)-based process was conducted with pathogen primer listed in **Supplementary Information Table S6**. DNA and RNA extraction were same as Probe-Capture Metagenomics. Then, the master mix including 2× RT PCR buffer, Primer mix and 25× RT PCR enzyme, was prepared and loaded onto a 96-Well Plate for the Applied Biosystems® 7500 (ThermoFisher Scientific), with each master mix on the plate having a corresponding PC (positive control) and NC. Referring to manufacturers’ operating instructions for use of the Applied Biosystems® 7500, the PCR program was performed, with programming steps for the thermocycler were as follows: 15 min at 50˚C, then 94˚C for 1min, followed by 40 cycles of 8 sec at 94˚C and 1 min at 60˚C. The level of fluorescence increased by generation of amplicons that reported as a cycle threshold (Ct) value, was proportional to the amount of pathogen nucleic acid contained in samples.

The experimental quality control was deemed qualified when the amplification curve of PC exhibited a significant exponential growth period with an eligible Ct value, whereas the NC lacked any specific amplification curve or Ct value. A positive result was determined if the Ct value of the sample within the positive Ct threshold indicated by the instructions, and the concentration was then inferred with reference to the standard curve, which was created using the PC concentration and Ct value.

**Supplementary Figures**

**
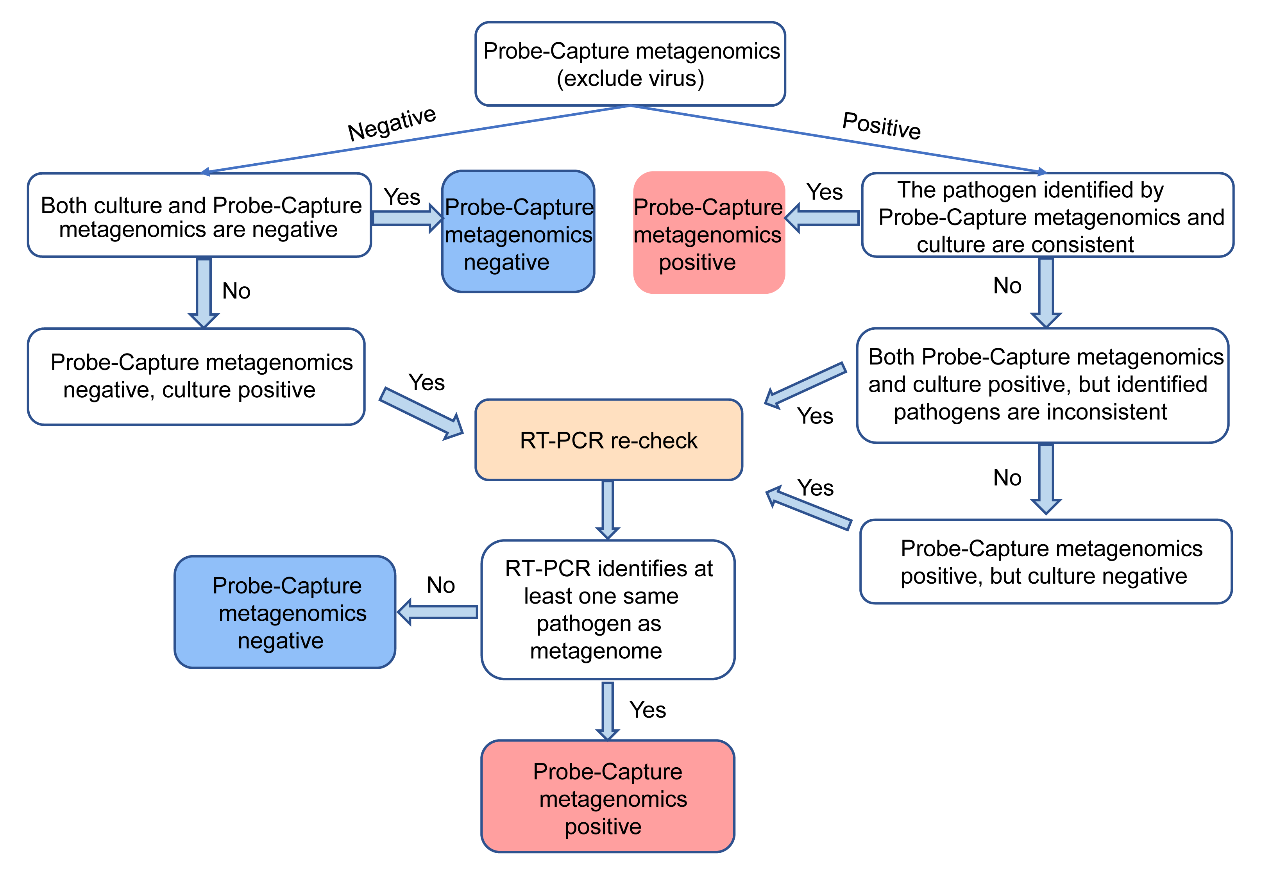
**

**Figure S1. Adjudication of the blood Probe-Capture Metagenomics result.**

**
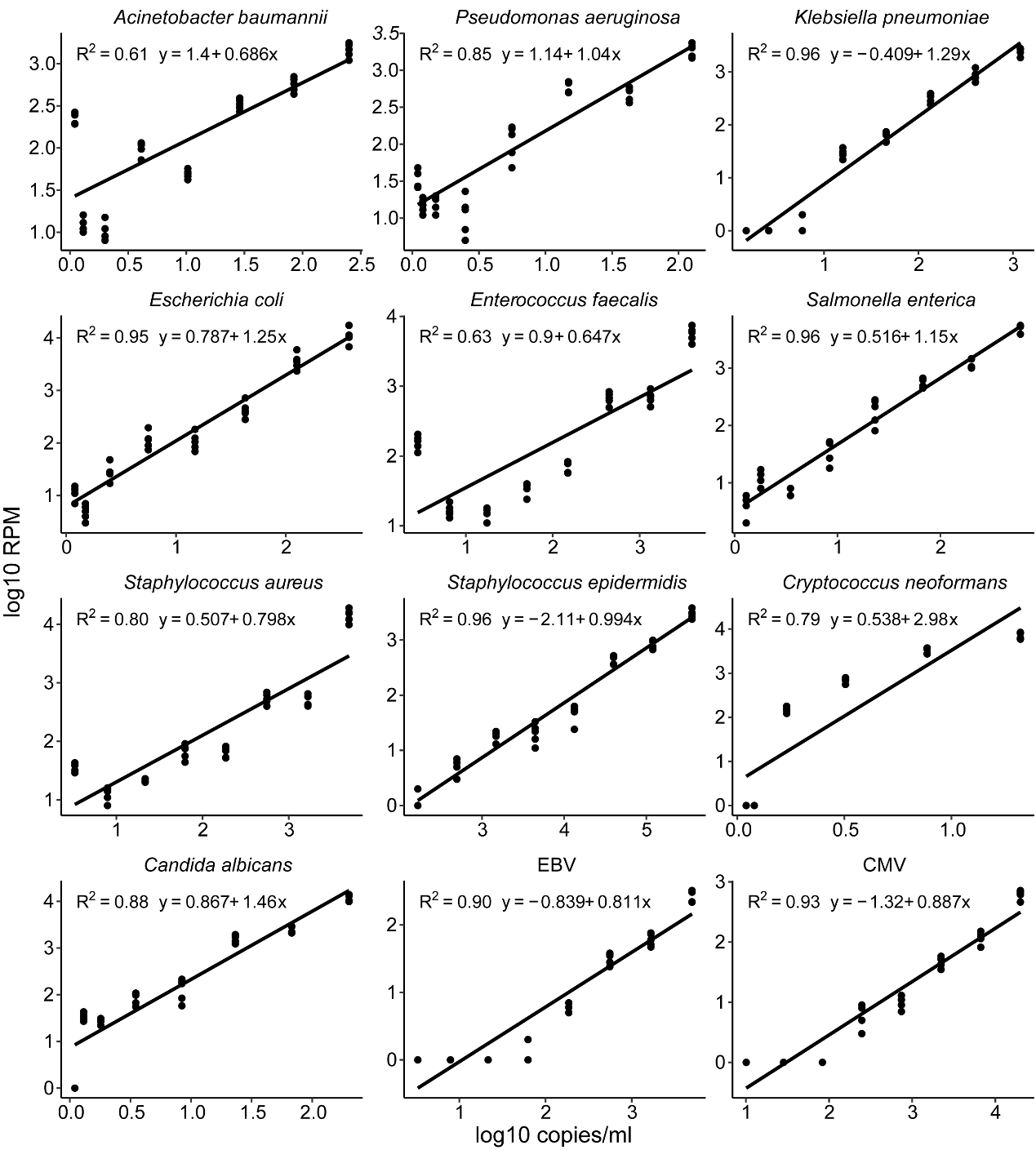
**

**Figure S2. Relationship of the microorganism concentration in HEK293T cells matrix with Probe-Capture Metagenomics detection signal [expressed in reads per million (RPM)].**

**
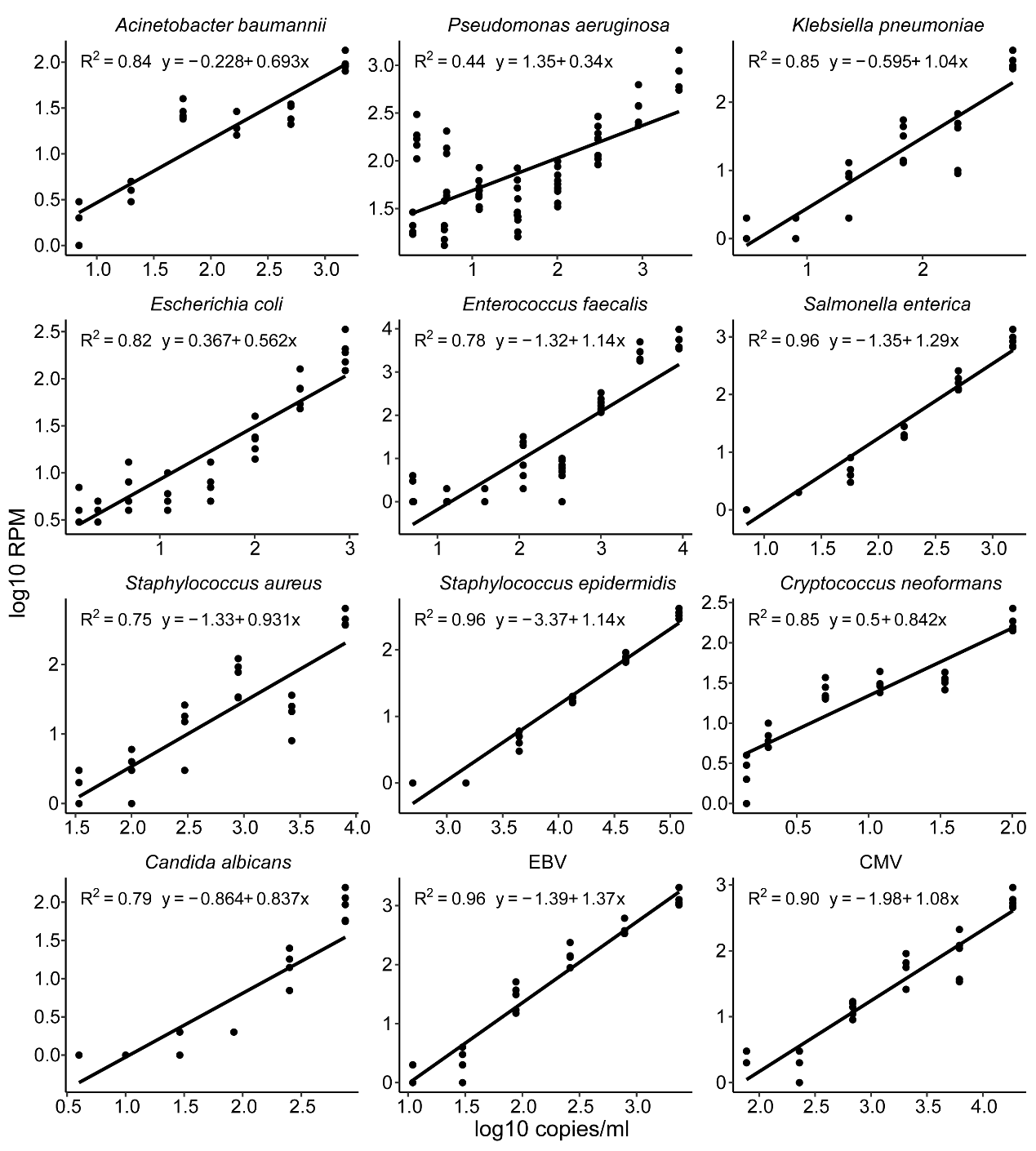
**

**Figure S3. Relationship of the microorganism concentration in plasma matrix with Probe-Capture Metagenomics detection signal [expressed in reads per million (RPM)]**


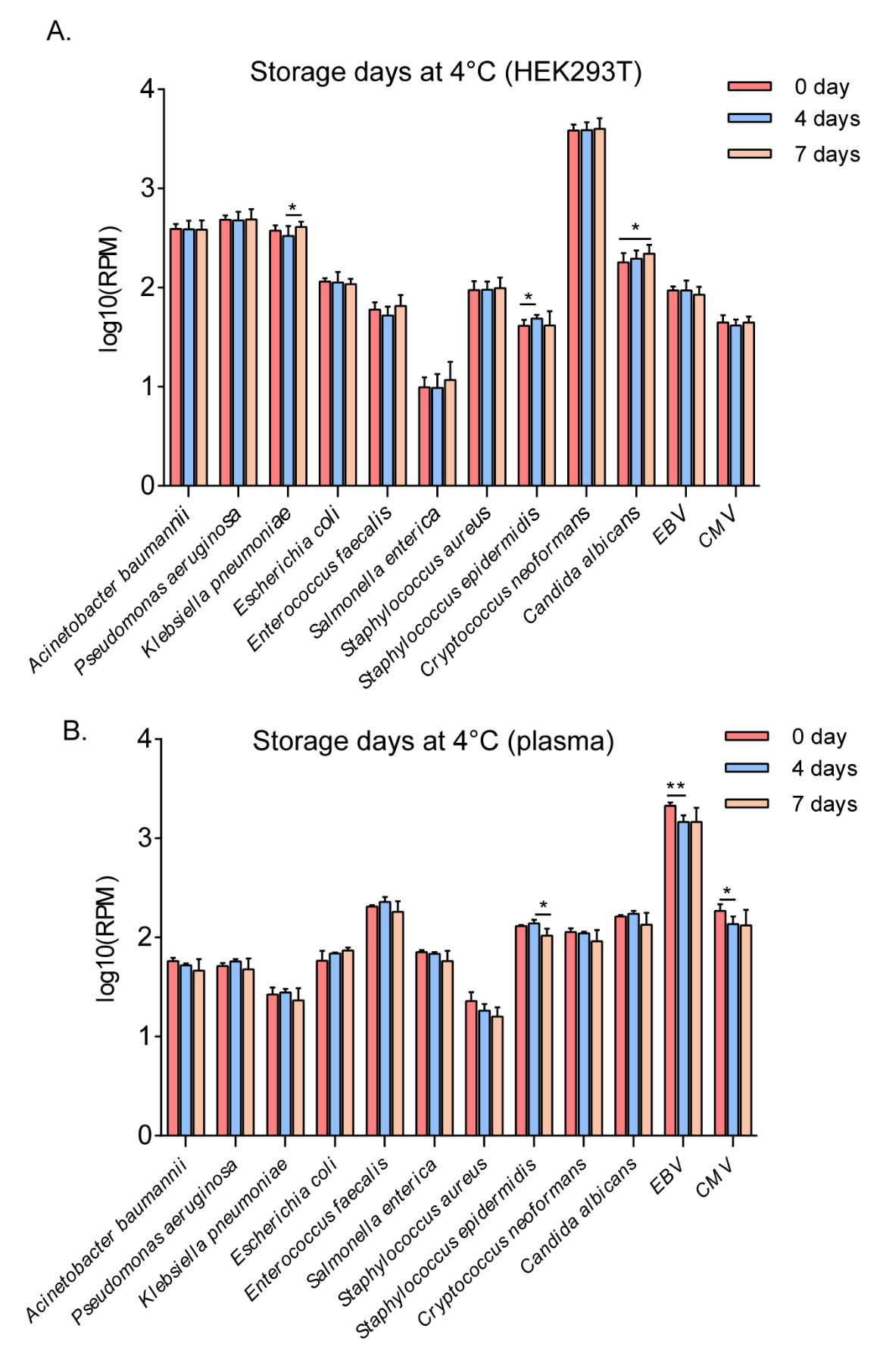


**Figure S4. Stability analysis of Probe-Capture Metagenomics assay.** The effect of storage duration at 4°C on pathogen detection diluted in HEK 293T cells matrix (A) and plasma matrix (B).

**
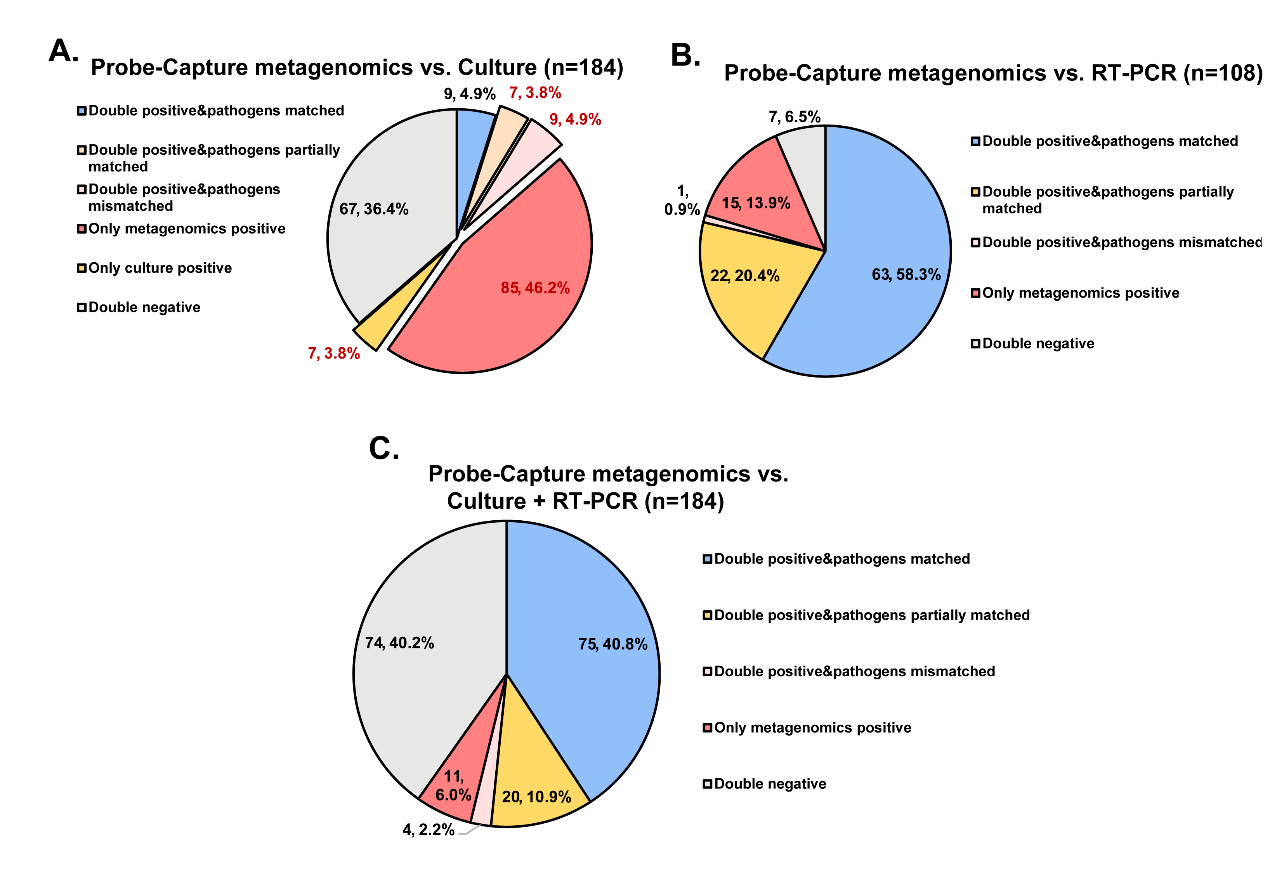
**

**Figure S5. The results consistency between Probe-Capture metagenome, culture and RT-PCR.** A. Comparison of the results and pathogens consistency between two groups by Probe-Capture metagenome and culture, red font represents inconsistent results that need to be validated by RT-PCR. B. Comparison of the results and pathogens consistency between two groups by Probe-Capture metagenome and RT-PCR for inconsistent samples in Figure A (red font). C. After combing the results of culture and RT-PCR, pie chart demonstrating the result consistency of Probe-Capture metagenome and culture + RT-PCR. Pathogens matched: detected pathogens were identical; Partially matched: at least one overlap of pathogens were observed; Mismatched: no overlap of detected pathogens.


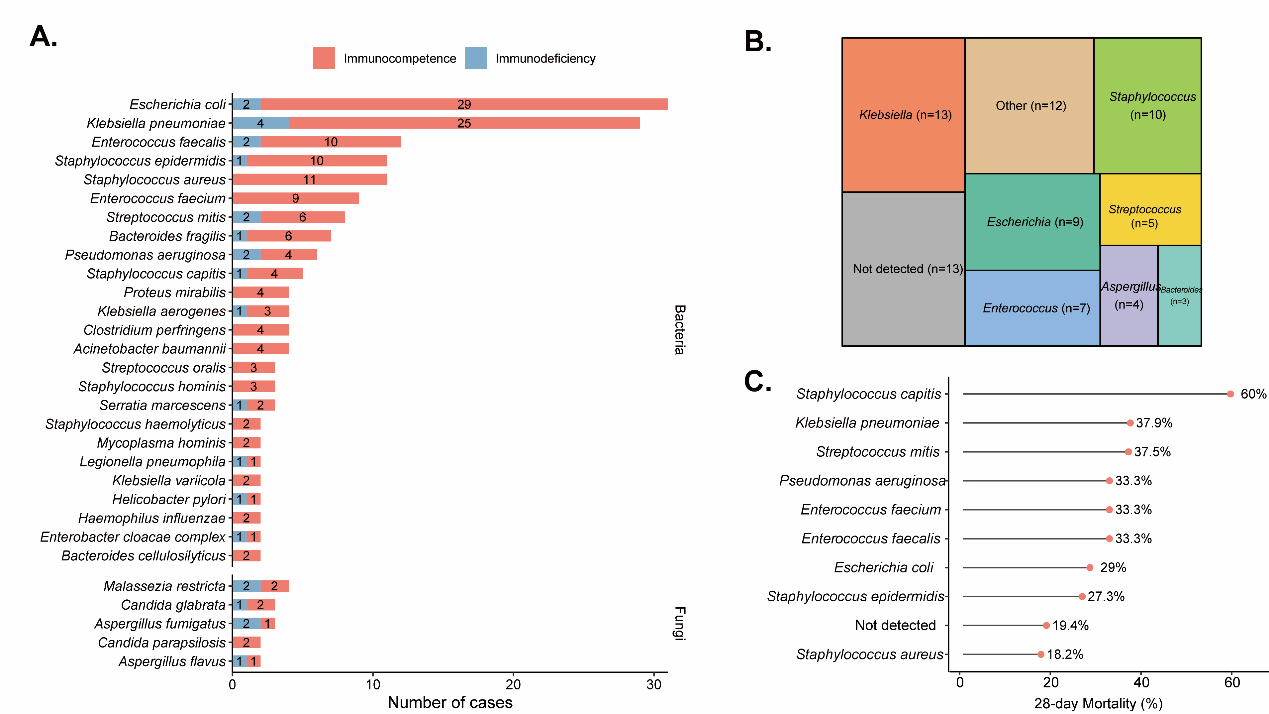


**Figure S6.** Distribution of detected pathogens and correlation to clinical outcome. A, Bacteria and fungi identified among patients with immunodeficiency and immunocompetence. Pathogens with a frequency greater than 1 are listed in order of the total detected frequency in all samples. B, The distribution of pathogens at genus level in 28-day non-survived patients (43 patients). C, The 28-day mortality of specific pathogens that detected by culture or Probe-Capture Metagenomics in corresponding patients.

**Supplementary Tables**

**Table S1. Comparison of patients’ characteristics between patients with positive impact and not.**

| **Characteristics** | **With positive impact** | **Without positive impact** | **P value** |
| --- | --- | --- | --- |
| **Number of patients** | 41 | 143 | / |
| **Sex, male** | 29 (70.73) | 95 (66.43) | 0.604763 |
| **Age, years** | 66 (55.5, 70) | 67 (56, 74) | 0.296859212 |
| **Medical history** |  |  |  |
| Hypertension | 22 (53.66) | 61 (42.66) | 0.212037 |
| Cerebral infarction | 9 (21.95) | 13 (9.09) | 0.051569 |
| Diabetes mellitus | 8 (19.51) | 33 (23.08) | 0.62871 |
| Coronary heart disease | 7 (17.07) | 20 (13.99) | 0.622374 |
| COPD | 3 (7.32) | 0 (0) | **0.010437** |
| Chronic cardiac insufficiency | 2 (4.88) | 5 (3.5) | 0.653117 |
| CKD | 3 (7.32) | 7 (4.9) | 0.695145 |
| Malignant tumor | 6 (14.63) | 10 (6.99) | 0.203523 |
| Autoimmune disease | 5 (12.2) | 11 (7.69) | 0.357002 |
| Immunodeficiency | 8 (19.51) | 24 (16.78) | 0.698255 |
| **Laboratory examination** |  |  |  |
| WBC-day0, 10^9^/L | 10.74 (5.6, 16.71) | 12.21 (8.08, 17.32) | 0.2909397 |
| WBC-day3, 10^9^/L | 8.64 (5.98, 14.2) | 10.29 (6.9, 14.95) | 0.391546203 |
| Neutrophil count-day0, 10^9^/L | 8.27 (4.3, 15.04) | 10.39 (6.46, 15.72) | 0.263028838 |
| Neutrophil count-day3,10^9^/L | 7.76 (4.79, 12.23) | 9 (5.61, 12.99) | 0.449403438 |
| CRP-day0, mg/L | 105.13 (64, 215.3) | 110.54 (44.45, 200.5) | 0.532478987 |
| CRP-day3, mg/L | 60.34 (35.1, 132.52) | 72.6 (29.81, 147.79) | 0.672219789 |
| PCT-day0, μg/L | 4.71 (0.9, 26.9) | 4.5 (0.74, 23.45) | 0.905412999 |
| PCT-day3, mg/L | 2.41 (0.32, 9.85) | 2.01 (0.59, 6.52) | 0.952285832 |
| **Severity and Outcome** |  |  |  |
| SOFA-day0 | 10 (8, 13) | 9 (6, 11) | **0.022993489** |
| SOFA-day3 | 8.5 (6, 12) | 6 (5, 10) | **0.021589613** |
| SOFA-day7 | 7 (4, 10) | 5 (2, 8) | 0.065240283 |
| Delta SOFA (day0-3) | 1.5 (0.25, 3) | 2 (0, 3) | 0.946735393 |
| Delta SOFA (day3-7) | 3 (1, 4) | 2 (0.5, 3) | 0.091282675 |
| Delta SOFA (day0-7) | 4 (2, 6) | 4 (1, 6) | 0.356533724 |
| Mechanical ventilation | 31 (75.61) | 96 (67.13) | 0.300752 |
| 28-day mortality | 14 (32.56) | 29 (20.28) | 0.06436 |
| Hospitalization mortality | 1 (2.33) | 9 (6.29) | 0.337154 |

COPD, chronic obstructive pulmonary disease; CKD, chronic renal disease; WBC, white blood cell; CRP, C-reactive protein; PCT, procalcitonin; SOFA, Sequential Organ Failure Assessment.

Continuous variables were presented as medians (first quartile, third quartile) and categorical variables as counts and percentages, unless otherwise specified.

**Table S2. Comparison of patients’ characteristics between patients received antibiotic adjustment and not.**

| Characteristics | Antibiotic adjustment | Antibiotic un-adjustment | P value |
| --- | --- | --- | --- |
| **Number of patients** | 64 | 120 | / |
| **Gender, male, n (%)** | 45 (70.31) | 79 (65.83) | 0.5370 |
| **Age, years** | 66 (56, 71) | 67 (56, 74) | 0.4602 |
| **Severity and Outcome** |  |  |  |
| SOFA-day0 | 10 (8, 13) | 9 (6, 11) | **0.0064** |
| SOFA-day3 | 8 (6, 12) | 6 (5, 10) | **0.0082** |
| SOFA-day7 | 7.5 (4, 11) | 4 (2, 8) | **0.0002** |
| Delta SOFA (day0-3) | 2 (1, 3) | 2 (0, 3) | 0.8011 |
| Delta SOFA (day3-7) | 1 (0, 3) | 2 (1, 3) | 0.0836 |
| Delta SOFA (day0-7) | 3 (2, 5) | 4 (1, 6) | 0.3633 |
| Mechanical ventilation | 45 (70.31) | 82 (68.33) | 0.7821 |
| 28-day mortality | 22 (34.38) | 21 (17.5) | **0.0100** |
| Hospitalization mortality | 3 (4.69) | 7 (5.83) | 0.7440 |
| **Laboratory examination** |  |  |  |
| WBC-day0, 10^9^/L | 10.13 (5.66, 16.41) | 12.39 (8.52, 17.34) | 0.0734 |
| WBC-day3, 10^9^/L | 8.8 (6.08, 13.67) | 10.61 (6.91, 15.12) | 0.1959 |
| Neutrophil count-day0, 10^9^/L | 7.99 (4.56, 14.62) | 11.09 (7.12, 16.35) | **0.0303** |
| Neutrophil count-day3, 10^9^/L | 7.9 (4.9, 11.74) | 9 (5.63, 13.33) | 0.2676 |
| CRP-day0, mg/L | 121.2 (64.35, 183.43) | 127.82 (49.67, 196.43) | 0.9884 |
| CRP-day3, mg/L | 71.21 (37.02, 115.22) | 95.82 (33.23, 141.87) | 0.2504 |
| PCT-day0, μg/L | 2.71 (0.79, 17.02) | 5.8 (0.78, 25.53) | 0.2694 |
| PCT-day3, mg/L | 1.31 (0.33, 6.62) | 2.81 (0.77, 8.69) | 0.2119 |

SOFA, Sequential Organ Failure Assessment; WBC, white blood cell; CRP, C-reactive protein; PCT, procalcitonin. Continuous variables were presented as medians (first quartile, third quartile) and categorical variables as counts and percentages, unless otherwise specified.

**Table S3.** Cost comparison between Traditional Metagenomics and Probe-Capture Metagenomics workflows

|  | **Traditional Metagenomics** | **Probe-Capture Metagenomics** |
| --- | --- | --- |
| **DNA/RNA extraction** | $15.00 | $15.00 |
| **cDNA reverse transcription and ribosomal removement** | $25.00 | $25.00 |
| **Library Construction** | $27.50 | $27.50 |
| **Probe Capture** | - | $130.00 |
| **NextSeq Sequencing** | $140.00 | $14.00 |
| **Total cost** | $207.50 | $211.50 |

**Table S4-S7 were uploaded separately as an excel file:**

Table S4. Clinical reportable pathogen and pathogen used for probe design.

Table S5. The list of accension ID of our database.

Table S6. Primer information of real time PCR for Probe-Capture Metagenomics validation.

Table S7. Clinical data of 184 patients.
